# Comparative Effectiveness of Phosphate Binders in Patients with Chronic Kidney Disease: A Systematic Review and Network Meta-Analysis

**DOI:** 10.1371/journal.pone.0156891

**Published:** 2016-06-08

**Authors:** Nigar Sekercioglu, Lehana Thabane, Juan Pablo Díaz Martínez, Gihad Nesrallah, Christopher J. Longo, Jason W. Busse, Noori Akhtar-Danesh, Arnav Agarwal, Reem Al-Khalifah, Alfonso Iorio, Gordon H. Guyatt

**Affiliations:** 1 Department of Clinical Epidemiology and Biostatistics, McMaster University, Hamilton, Ontario, Canada; 2 Department of Pediatrics and Anesthesia, McMaster University, Hamilton, Ontario, Canada; 3 Centre for Evaluation of Medicine, St Joseph's Healthcare, Hamilton, Ontario, Canada; 4 Biostatistics Unit, Father Sean O'Sullivan Research Centre, St Joseph's Healthcare, Hamilton, Ontario, Canada; 5 Population Health Research Institute, Hamilton Health Sciences, Hamilton, Ontario, Canada; 6 Humber River Hospital, Toronto, Ontario, Canada; 7 Department of Medicine, University of Toronto, Toronto, Ontario, Canada; 8 DeGroote School of Business, 4350 South Service Road, Burlington, Ontario, Canada; 9 The Michael G. DeGroote Institute for Pain Research and Care, McMaster University, Hamilton, Ontario, Canada; 10 Department of Anesthesia, McMaster University, Hamilton, Ontario, Canada; 11 Faculty of Medicine, University of Toronto, Toronto, Ontario, Canada; 12 Division of Pediatric Endocrinology, King Saud University, Riyadh, Saudi Arabia; 13 Department of Medicine, McMaster University, Hamilton, Ontario, Canada; Sao Paulo State University, BRAZIL

## Abstract

**Background:**

Chronic kidney disease-mineral and bone disorder (CKD-MBD) has been linked to poor health outcomes, including diminished quality and length of life. This condition is characterized by high phosphate levels and requires phosphate-lowering agents—phosphate binders. The objective of this systematic review is to compare the effects of available phosphate binders on patient-important outcomes in patients with CKD-MBD.

**Methods:**

Data sources included MEDLINE and EMBASE Trials from 1996 to February 2016. We also searched the Cochrane Register of Controlled Trials up to April 2016. Teams of two reviewers, independently and in duplicate, screened titles and abstracts and potentially eligible full text reports to determine eligibility, and subsequently abstracted data and assessed risk of bias in eligible randomized controlled trials (RCTs). Eligible trials enrolled patients with CKD-MBD, randomized them to receive calcium (delivered as calcium acetate, calcium citrate or calcium carbonate), non-calcium-based phosphate binders (NCBPB) (sevelamer hydrochloride, sevelamer carbonate, lanthanum carbonate, sucroferric oxyhydroxide and ferric citrate), phosphorus restricted diet, placebo or no treatment, and reported effects on all-cause mortality, cardiovascular mortality or hospitalization at ≥4 weeks follow-up. We performed network meta-analyses (NMA) for all cause-mortality for individual agents (seven-node analysis) and conventional meta-analysis of calcium vs. NCBPBs for all-cause mortality, cardiovascular mortality and hospitalization. In the NMAs, we calculated the effect estimates for direct, indirect and network meta-analysis estimates; for both NMA and conventional meta-analysis, we pooled treatment effects as risk ratios (RR) and calculated 95% confidence intervals (CIs) using random effect models. We used the GRADE (Grading of Recommendations, Assessment, Development and Evaluation) approach to rate the quality of evidence for each paired comparison.

**Results:**

Our search yielded 1190 citations, of which 71 RCTs were retrieved for full review and 15 proved eligible. With 13 eligible studies from a prior review, we included 28 studies with 8335 participants; 25 trials provided data for our quantitative synthesis. Results suggest higher mortality with calcium than either sevelamer (NMA RR, 1.89 [95% CI, 1.02 to 3.50], moderate quality evidence) or NCBPBs (conventional meta-analysis RR, 1.76 [95% CI, 1.21 to 2.56, moderate quality evidence). Conventional meta-analysis suggested no difference in cardiovascular mortality between calcium and NCBPBs (RR, 2.54 [95% CI, 0.67 to 9.62 low quality evidence). Our results suggest higher hospitalization, although non-significant, with calcium than NCBPBs (RR, 1.293 [95% CI, 0.94 to 1.74, moderate quality evidence).

**Discussion/Conclusions:**

Use of calcium results in higher mortality than either sevelamer in particular and NCBPBs in general (moderate quality evidence). Our results raise questions about whether administration of calcium as an intervention for CKD- MBD remains ethical. Further research is needed to explore the effects of different types of phosphate binders, including novel agents such as iron, on quality and quantity of life.

**Systematic Review Registration:**

PROSPERO CRD-42016032945

## Introduction

Patients with chronic kidney disease (CKD) [1] are at higher risk of death, often due to cardiovascular disease [2–7]. CKD leads to hyperphoshatemia and a number of chronic disturbances of calcium-phosphate homeostasis collectively referred to as CKD mineral and bone disorder (CKD-MBD). This constellation of metabolic abnormalities leads to arterial intimal and medial calcification that are associated with cardiovascular events [2], while abnormal bone turnover, architecture and mineralization result in reduced bone quality and density, with increased risk of fracture [2].

Phosphate has long been considered an important target for managing CKD-MBD and its sequelae. Because of the adverse impact of high serum phosphate levels on cardiovascular and bone outcomes and on survival [8–11], and because elevated serum phosphate is common in CKD patients, phosphate binders have a pivotal role in the management of CKD. Calcium—delivered as calcium acetate, calcium citrate or calcium carbonate—is less expensive, but more likely to cause hypercalcemia [8–11]. Non-calcium-based phosphate binders (NCBPB), sevelamer and lanthanum, are costlier but do not cause hypercalcemia [8–11].

Through different mechanisms, all phosphate binders prevent phosphate absorption from the gastrointestinal system [12]. Sevelamer is a resin-based binder with an anion exchange mechanism [13]. Lanthanum binds phosphate through its trivalent cation [13]. Recently, iron (e.g., ferric citrate and sucroferric oxyhydroxide) has also proved effective in lowering phosphate by impeding the absorption of phosphate in the stomach without evidence of toxicity [14,15]. The crucial question, however, is the relative impact of these agents on patient-important outcomes, particularly on mortality.

Jamal et al. conducted a meta-analysis of 15 randomized control trials (RCTs) examining CBPBs versus NCBPBs in patients with hyperphosphatemia and CKD. The results suggest higher mortality with CBPBs than with NCBPBs[16]. Inferences from this review are limited because the review did not address individual NCBPBs and because of imprecision of the main finding: results were consistent with either a moderate relative reduction in mortality (23%) or a very small relative reduction (3%). Moreover, the quality appraisal was limited, reducing overall confidence in the estimates of effect and conclusions [16].

The objective of this systematic review was (1) to update the Jamal et al. systematic review[16] using the Grading of Recommendations, Assessment, Development and Evaluation (GRADE) approach and (2) to provide estimates of effect of individual agents by combining direct and indirect estimates through a network meta-analysis (NMA).

## Methods

We registered our protocol with PROSPERO (http://www.crd.york.ac.uk/PROSPERO/display_record.asp?ID=CRD42016032945). We adhered to the PRISMA NMA guidelines in drafting our manuscript (http://www.prisma-statement.org/Extensions/NetworkMetaAnalysis.aspx) (S1 File).

### Eligibility criteria

We included studies that (1) enrolled adult patients (≥18 years of age) with chronic kidney disease, defined as an estimated glomerular filtration rate <60 ml/min/1.73 m^2^, including dialysis CKD patients (CKD stage 5D) and non-dialysis CKD patients (stages 3 through 5) [1, 17]; (2) randomized patients to a phosphate binder or a control. Phosphate binders included CBPBs (calcium acetate, calcium citrate or calcium carbonate) and NCBPDs (sevelamer hydrochloride, sevelamer carbonate, lanthanum carbonate, sucroferric oxyhydroxide or ferric citrate). A control included phosphorus restricted diet, placebo or no intervention; (3) reported at least one of the following outcomes: all-cause mortality, cardiovascular mortality or hospitalization due to any cause; and (4) had a minimum follow-up of 4 weeks. We excluded studies that included pediatric patients if outcomes of adults were not reported separately.

### Data sources and search strategy

We included all trials identified in a prior review and updated the search for the subsequent period [16]; specifically, we searched MEDLINE and EMBASE from January 2013 until February 2016 without language restrictions. We also searched the Cochrane Register of Controlled Trials up to April 2016. We used controlled vocabulary and text words and restricted our search to RCTs. We scanned the bibliographies of all prior systematic reviews and meta-analyses as well as all eligible primary studies for additional relevant articles. Our full search strategy is depicted in S2 File in supporting information.

### Study selection

Teams of two reviewers independently screened each title and abstract. If either reviewer identified a citation as potentially relevant, we obtained the full text of the article. Two reviewers independently determined the eligibility of all studies that underwent full text evaluation. If we found more than one publication for a study, and if supplementary reports included eligible outcome measures not provided in the main report, we included complementary information from the second or third report.

### Data abstraction

We extracted study data using a customized data collection form accompanied by a detailed instruction manual. We abstracted the following information from each study: author, year of publication, baseline characteristics of participants, number of participants in each arm at study onset and completion, trial duration and treatment effects. We recorded the last measurement if multiple measurements were provided during the follow-up period.

### Risk of bias of included studies

Two independent reviewers used a modified version of the Cochrane risk for bias tool in order to assess the risk of bias on the basis of randomization, allocation concealment, blinding, incomplete outcome data, selective reporting (by comparing the methods and results sections of the manuscript) as well as stopping early for benefit [18]. Reviewers chose among response options of “definitely yes”, “probably yes”, “probably no”, and “definitely no” for each of the domains, with “definitely yes” and “probably yes” ultimately assigned low risk of bias and “definitely no” and “probably no” assigned high risk of bias [19]. For eligibility and risk of bias, reviewers resolved disagreements by discussion.

### Quality assessment of bodies of evidence

#### Quality assessment of direct evidence

We assessed the quality of evidence in effect estimates for each outcome as high, moderate, low or very low using the GRADE rating system [20]. In the GRADE system, RCTs begin as high quality evidence, but may be rated down by one or more of five categories of limitations [19]: risk of bias, precision, consistency, directness and publication bias [21].

Clinical heterogeneity was assessed in terms of differences in population, intervention, outcomes and settings (primary vs secondary vs tertiary care settings) and was used to judge directness. Statistical heterogeneity was assessed by visual inspection of forest plots for the degree of proximity in point estimates and overlap in 95% confidence intervals (95% CIs) and by the Chi-Square test of homogeneity, and the I^2^ statistic for which 0–40% may be unimportant heterogeneity, 30–60% moderate, 50–90% substantial and 75–100% considerable heterogeneity [22].

With respect to precision, we assessed the width of the 95% CIs for inclusion of values that would alter clinical decision-making [23]. Publication bias was considered undetected unless the effect measure was asymmetrically distributed around the pooled effect [24, 25].

After considering these reasons for rating down, we judged the overall confidence in estimates of effect for all-cause mortality, cardiovascular mortality and hospitalization for each direct comparison as follows: ‘high’ quality of evidence (we are very confident that the true effect lies close to that of the estimate of the effect); ‘moderate’ quality of evidence (we are moderately confident in the effect estimate and the true effect is likely to be close to the estimate of the effect, but there is a possibility that it is substantially different); ‘low’ quality of evidence (our confidence in the effect estimate is limited and the true effect may be substantially different from the estimate of the effect); and ‘very low’ quality of evidence (we have very little confidence in the effect estimate and the true effect is likely to be substantially different from the estimate of effect) [19].

#### Quality assessment of indirect evidence

We also applied the GRADE methodology to rate the confidence of indirect effect estimates. Indirect effect estimates are calculated from available ‘loops’ of evidence, which includes first order (based on a single common comparator treatment, the difference between the treatment A and B is based on comparisons of A and C as well as B and C, as with ${\hat{d}}_{AB}^{I}\;=\;{\hat{d}}_{AC}^{D}-{\hat{d}}_{BC}^{D}$) or higher order (more than one intervening treatment connecting the two interventions that constitute the comparison of interest) [26].

To judge the quality of the indirect comparison we chose the first order loop with the lowest variance in those without a common comparator. The quality of evidence rating for indirect comparisons was the lower of the ratings of quality for the two direct estimates that contribute to the first order loop of the indirect comparison. For instance, if one of the direct comparison was rated as low and other was rated as moderate evidence, we rated the quality of indirect evidence as low [27].

We also considered further rating down the quality of the indirect comparison for intransitivity. The transitivity assumption implies similarity of trials in terms of population, intervention (type and dosing frequency), settings and trial methodology. If the transitivity assumption was violated, we rated down indirect comparison one further level.

#### Quality assessment of NMA mixed estimates

If both direct and indirect evidence were available, the NMA mixed estimate quality rating came from the higher quality of the two. We also considered coherence (degree of consistency between direct and indirect effect estimates) in our final quality rating. We examined the magnitude of the difference between direct and indirect effect estimates and the extent to which confidence intervals overlapped and rated down confidence the quality of the NMA effect if we found large incoherence defined as inconsistency between direct and indirect effect estimates.

Asymmetrical funnel plots indicate reporting biases due to publication bias or small study effect [24]. We employed the comparison-adjusted funnel plot using fixed effect models. The black dashed line indicates the estimated small-study effects line—also called the regression line.

Thus, the quality of evidence for each paired network comparison included assessment of transitivity (similarity between populations, interventions, comparators and outcomes of trials in the direct comparisons that contribute to the indirect comparison estimate); coherence (similarity between direct and indirect effects); and homogeneity (similarity of effect estimates between trials in direct comparisons).

### Data synthesis and statistical analysis

For conventional meta-analyses (all individual paired comparisons and comparison of calcium versus NCBPBs for all-cause and cardiovascular mortality, and for hospitalization) we calculated risk rations (RRs) and 95% confidence intervals (CIs) using random effects models. For our NMA, we synthesised the results from RCTs using the frequentist approach. The relevant analysis was a seven-node network meta-analysis (NMA) (sevelamer hydrochloride vs. calcium carbonate vs. lanthanum carbonate vs. iron vs. phosphorus restricted diet vs. placebo vs. sevelamer-plus-calcium-plus-magnesium). We report pooled RRs for direct, indirect and mixed network meta-analysis estimates and associated 95% CIs. We present the direct, indirect and network effect estimates. We summarized the overall network heterogeneity using the global test [28]. We used the inconsistency factor for the assessment of loop inconsistency in our triangular loop [28–30]. The contribution plot indicates the contribution of each direct comparison to indirect and network estimates [28].

To estimate absolute benefit for statistically significant mortality benefit we used the median baseline risk of all studies with a calcium arm and applied the relative effect from the NMA mixed comparisons. We performed all analyses with Stata (StataCorp. 2013. Stata Statistical Software: Release 13. College Station, TX: StataCorp LP) using the **mvmeta** command.

## Results

### Trial identification

**Fig 1:**
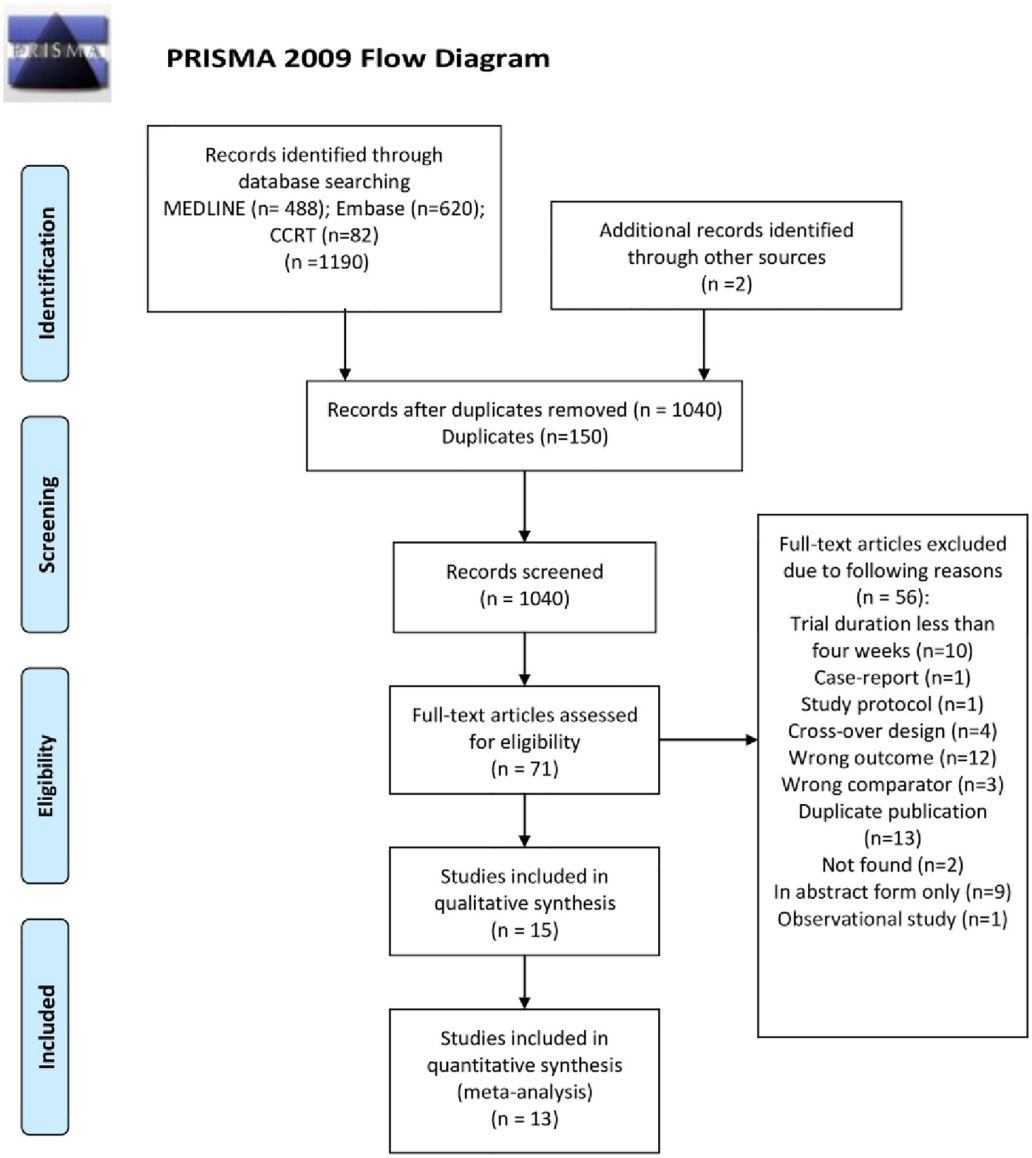
PRISMA Flow Diagram of Search Results. *From*: Moher D, Liberati A, Tetzlaff J, Altman DG, The PRISMA Group (2009). *P*eferred *R*eporting *I*terns tor *S*ystematic Reviews and *M*ela-*A*nalyses: The PRISMA Statement. PLoS Med 6(6): e1000097. doi:10.1371/journal.pmed1000097 For more information, visit www.prisma-statement.org.

Our updated search yielded 1190 citations, of which 71 were retrieved for full review; 15 RCTs proved eligible with 3576 (Fig 1). We included 13 RCTs from the previous systematic review [16]. Therefore, we included a total of 28 studies with 8335 participants; 25 provided data that allowed inclusion in our quantitative synthesis (Fig 1).

### Trial and population characteristics

S1 Table in the supporting information presents the characteristics of all eligible studies, of which 25 reported all-cause mortality [31–58]. Seven of the 28 studies (25%) included non-dialysis patients. Year of publication ranged from 2002 to 2015. Most of the trials were multinational (11 studies) and all were multi-centre. The mean age of participants ranged from 47 to 69.

**Fig 2:**
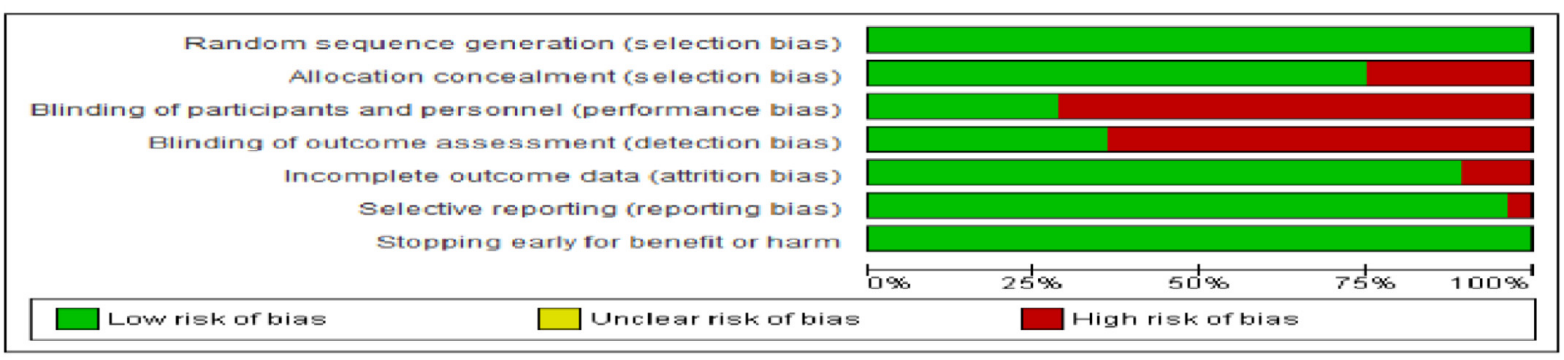
Risk of bias assessment; outcome: all-cause mortality. Legend: Our assessment indicated low risk of bias for missing data in about 75% of trials. The level of blinding was adequate in only about 25% of the studies.

Our assessment indicated low risk of bias for missing data and selective reporting in about 75% of the trials; blinding was adequate in only about 25% of the studies (Fig 2 and S1 Fig in the supporting information).

### Seven-node analysis

**Fig 3:**
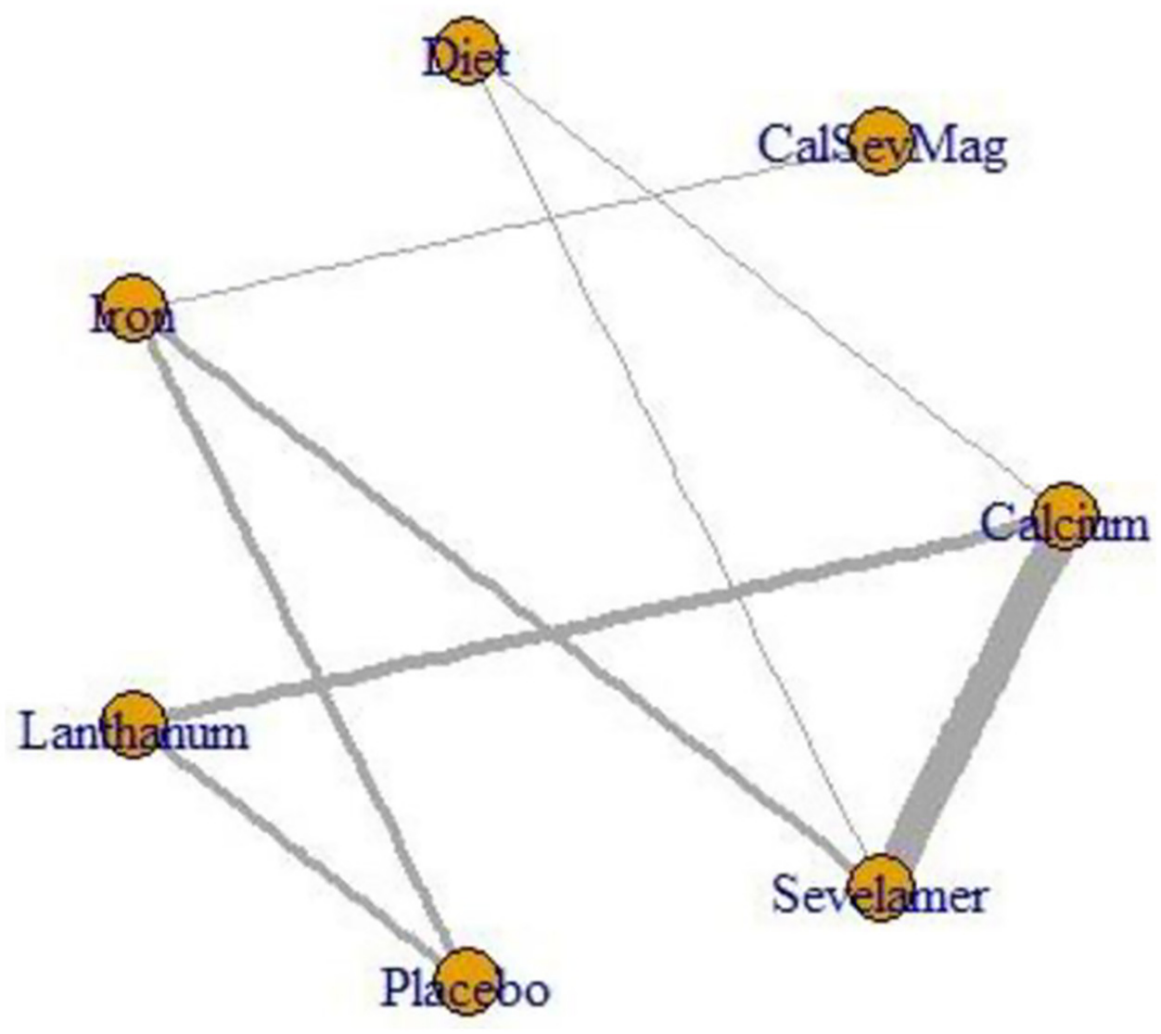
The network map of seven-node analysis; outcome: all-cause mortality. Legend: Edges are weighted by precision.

Fig 3 presents the network geometry of all-cause mortality and provides 8 direct and 13 indirect comparisons for seven interventions: sevelamer, lanthanum, iron, calcium, phosphorus restricted diet, sevelamer-plus-calcium-plus-magnesium and placebo. One trial compared three treatments [59]. Pairwise comparisons demonstrated I^2^ values from 0% to 81.6% (Table 1).

**Table 1:** GRADE quality assessment of direct evidence of each pairwise treatment comparison for all-cause mortality.

Treatment comparison	Number of head-to-head trials; n	Study Limitations	Precision	Consistency	Directness	Publication bias	Overall quality of evidence	Direct estimate^2^; RR (95% CI)	Absolute effect per 100 treated (95% CI)
Sevelamer vs.Calcium	10;3665	Not serious	Not serious	Serious (I^2^, 81.6%)	Not serious	Not serious	Moderate	1.89 (1.02 to 3.50)	43 cases more (23 more to 80 more)
Sevelamer vs. Iron	3; 1303	Serious (due to allocation concealment)	Serious	Not serious (I^2^, 0%)	Not serious	Not serious	Low	1.24 (0.48 to 3.18)	28 cases more (11less to 73 more)
Sevelamer vs. diet	1; 60	Not serious	Very serious^1^	Not serious	Not serious	Not serious	Low	0.33 (0.01 to 7.87)	8 cases less (1less to 181 more)
Lanthanum vs. Calcium	4; 1494	Serious (due to allocation concealment)	Not serious	Not serious (I^2^, 0%)	Not serious	Not serious	Moderate	1.17 (0.96 to 1.43)	27 cases more (22 less to 33 more)
Lanthanum vs Placebo	3; 408	Not serious	Very serious^1^	Not serious (I^2^, 0%)	Not serious	Not serious	Low	0.92 (0.11 to 7.31)	21 cases less (3 less to 168 more)
Calcium vs diet	1; 60	Not serious	Very serious^1^	Not serious (I^2^, 0%)	Not serious	Not serious	Low	0.33 (0.01 to 7.87)	8 cases less (1 less to 181more)
Iron vs. placebo	3; 561	Not serious	Very serious^1^	Not serious (I^2^, 0%)	Not serious	Not serious	Low	3.04 (0.40 to 23.31)	64 cases more (9 less to 529 more)
Iron vs. Sevelamer-plus-calcium-magnesium	1; 441	Not serious	Serious	Not serious	Not serious	Not serious	Moderate	0.81 (0.35 to 1.87)	19 less (8 cases less to 43 more)

For domains “Study Limitations”, “Precision”, “Consistency”, and “Directness”: Not serious, Serious, or Very serious issues. For the domain “Publication bias”: Not likely or Likely to exist. Reasons are provided when rating down. All direct comparisons begin with a “High” rating.

^1^Rated down two levels for imprecision;

^2^We employed random effect models.

CI: Confidence interval; RR: Risk ratio.

**Fig 4:**
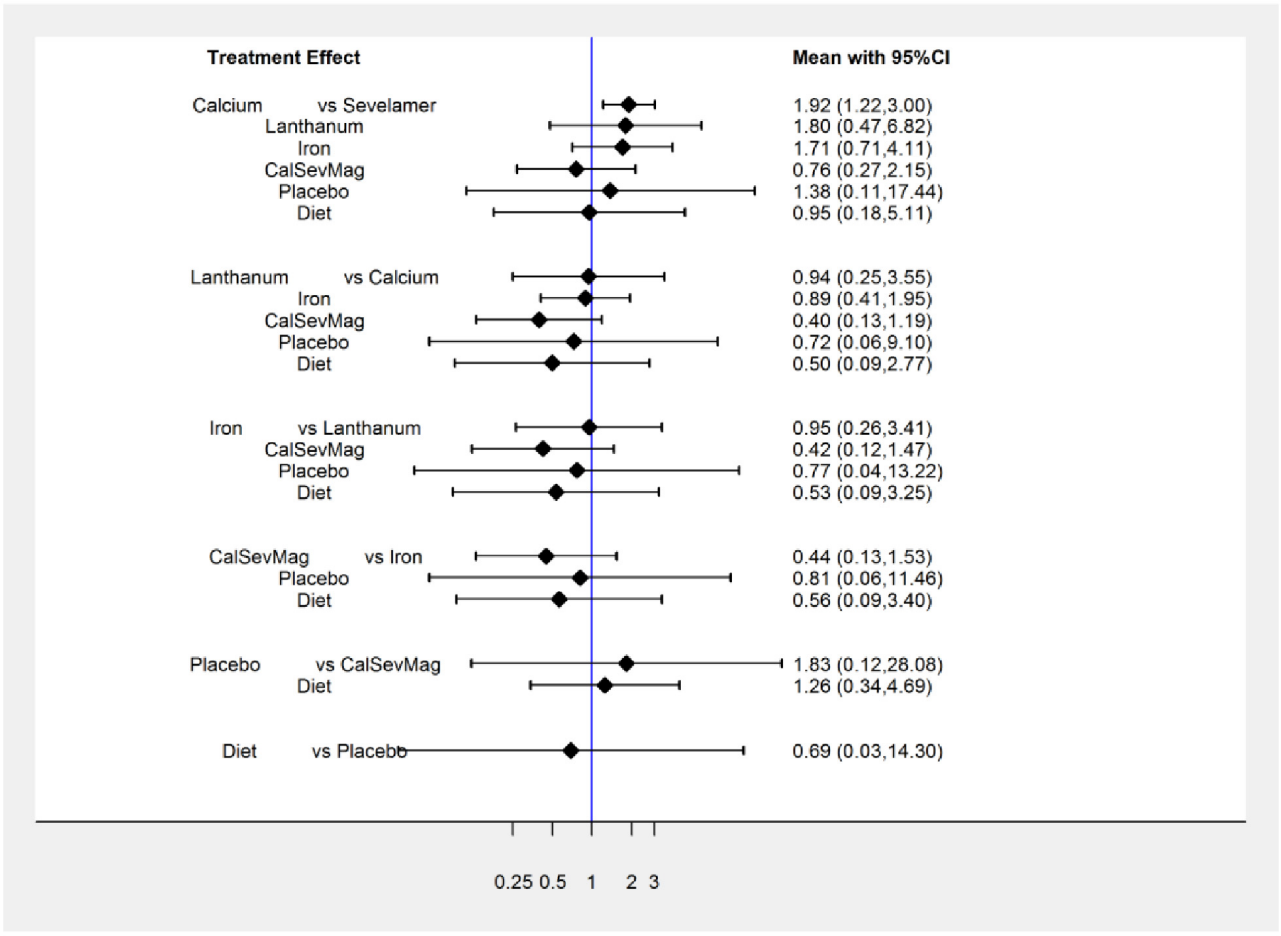
The predictive interval consistency plot from the consistency model of seven-node analysis; outcome: all-cause mortality without a reference standard.

For the seven-node comparison, Table 1 presents direct comparisons that contributed to the NMA, Table 2 the indirect comparisons with the associated quality of evidence ratings, and Table 3 the summary of results and quality of evidence. Moderate quality of evidence suggests higher mortality with calcium versus sevelamer (NMA RR, 1.89 [95% CI, 1.02 to 3.50]). Given a baseline mortality of 23% over a year this relative effect translates into an absolute mortality increase with calcium of 43 per 1000 (95% CI 23 to 80 more. Confidence intervals for all other comparisons included no effect. Fig 4 presents the confidence interval plot. S2 Fig in the supporting information depicts the contribution plot indicating the contribution of each direct comparison to indirect and network estimates.

**Table 2:** GRADE confidence assessments of indirect estimates per pairwise treatment comparison for all-cause mortality.

	Treatment comparisons	Common comparator treatment in thedominant first order loop (in the absence of the first order loop, higher order loop with the lowest variance)	GRADE of first contributing direct comparison	GRADE of second contributing direct comparison	Assessment of transitivity	Final GRADE of Indirect Comparison
1	Sevelamer vs. placebo	Iron	Low (sevelamer vs. iron)	Low (placebo vs. iron)	Not serious	Low
2	Sevelamer vs. Lanthanum	Calcium	Moderate (sevelamer vs. calcium)	Moderate (lanthanum vs. calcium)	Not serious	Moderate
3	Sevelamer vs. sevelamer plus calcium plus magnesium	Iron	Low (sevelamer vs. iron)	Moderate (iron vs. sevelamer-plus-calcium)	Not serious	Low
4	Calcium vs. placebo	Lanthanum	Moderate (calcium vs. lanthanum)	Low (lanthanum vs. placebo)	Not serious	Low
5	Calcium vs. Iron	Lanthanum placebo	Moderate (calcium vs. lanthanum)	Low (placebo vs. iron)	Not serious	Low
6	Calcium vs. sevelamer plus calcium plus magnesium	Lanthanum placebo	Moderate (calcium vs. lanthanum)	Low (placebo vs. iron)	Not serious	Low
7	Placebo vs. diet	Lanthanum placebo	Low (Calcium vs.diet)	Low (Lanthanum vs. placebo)	Not serious	Low
8	Placebo vs. sevelamer plus calcium plus magnesium	Iron	Low (placebo vs iron)	Moderate (Iron vs. sevelamer plus calcium)	Not serious	Low
9	Lanthanum vs. Iron	Placebo	Low (iron vs. placebo)	Low (lanthanum vs. placebo)	Not serious	Low
10	Lanthanum vs. diet	Calcium	Moderate (calcium vs. lanthanum)	Low (calcium vs. diet)	Not serious	Low
11	Lanthanum vs. sevelamer plus calcium plus magnesium	Lanthanum placebo	Moderate (calcium vs. lanthanum)	Low (placebo vs. iron)	Not serious	Low
12	Iron vs. diet	Sevelamer	Low (sevelamer vs. diet)	Low (sevelamer vs.iron)	Not serious	
13	Diet vs. sevelamer plus calcium plus magnesium	Lanthanum placebo	Low (Lanthanum vs. placebo)	Low (placebo vs. iron)	Not serious	Low

A single first order loop for each pairwise comparison is used to GRADE indirect estimates. All indirect comparisons begin with the lower of the two contributing direct estimates and undergo an assessment of transitivity. For the transitivity assumption: Not serious or serious to exist.

**Table 3:** Direct, indirect, and NMA estimates of all-cause mortality with 95% confidence intervals and GRADE assessments for each pairwise comparison within the network of seven phosphate binders.

	Comparison	Direct estimate; RR (95% CI)	Quality of evidence	Indirect estimate; RR (95% CI)	Quality of evidence	NMA estimate; RR (95% CI)	Quality of evidence
1	Placebo vs. sevelamer	Not available	Not available	1.38(0.11 to 17.44)	Low	1.38(0.11 to 17.44)	Low
2	Lanthanum vs. sevelamer	Not available	Not available	1.80 (0.47 to 6.82)	Moderate	1.80 (0.47 to 6.82)	Moderate
3	CalSevMag vs. sevelamer	Not available	Not available	0.76 (0.27 to 2.15)	Low	0.76 (0.27 to 2.15)	Low
4	Placebo vs. calcium	Not available	Not available	0.72 (0.06 to 9.10)	Low	0.72 (0.06 to 9.10)	Low
5	Iron vs. Calcium	Not available	Not available	0.89 (0.41 to 1.95)	Low	0.89 (0.41 to 1.95)	Low
6	CalSevMag vs. calcium	Not available	Not available	0.40 (0.13 to 1.19)	Low	0.40 (0.13 to 1.19)	Low
7	Diet vs. placebo	Not available	Not available	0.69 (0.03 to 14.3)	Low	0.69 (0.03 to 14.3)	Low
8	Placebo vs. CalSevMag	Not available	Not available	1.83 (0.12 to 28)	Low	1.83 (0.12 to 28)	Low
9	Iron vs. lanthanum	Not available	Not available	0.95 (0.26 to 3.41)	Low	0.95 (0.26 to 3.41)	Low
10	Diet vs. lanthanum	Not available	Not available	0.53 (0.09 to 3.25)	Low	0.53 (0.09 to 3.25)	Low
11	CalSevMag vs. lanthanum	Not available	Not available	0.42 (0.12 to 1.47)	Low	0.42 (0.12 to 1.47)	Low
12	Diet vs. iron	Not available	Not available	0.56 (0.09 to 3.4)	Low	0.56 (0.09 to 3.4)	Low
13	Diet vs. CalSevMag	Not available	Not available	1.26 (0.34 to 4.69)	Low	1.26 (0.34 to 4.69)	Low
14	Calcium vs. sevelamer	1.89 (1.02 to 3.50)	Moderate	0.51 (0.03 to 9.89)	Moderate	1.35 (1.14 to 1.60)	Low^1^
15	Iron vs. sevelamer	1.24 (0.48 to 3.18)	Low	0.81 (0.05–11.94)	Low	1.71 (0.71 to 4.11)	Very low^1^
16	Diet vs. sevelamer	0.33 (0.01 to 7.87)	Low	0.73 (0.23 to 2.35)	Low	0.95 (0.18 to 5.11)	Low
17	Lanthanum vs. Calcium	1.17 (0.96 to 1.43)	Moderate	1.03 (0.17 to 6.33)	Moderate	0.94 (0.25 to 3.55)	Moderate
18	Placebo vs. lanthanum	0.92 (0.11 to 7.31)	Low	0.50 (0.02 to 16.08)	Low	0.77 (0.04 to 13.22)	Low
19	Diet vs.calcium	0.33 (0.01 to 7.87)	Low	0.47 (0.07 to 2.96)	Low	0.50 (0.09 to 2.77)	Low
20	Placebo vs.iron	3.04 (0.40 to 23.31)	Low	0.56 (0.03 to 12.24)	Low	0.81 (0.06 to 11.46)	Low
21	CalSevMag vs. Iron	0.81 (0.35 to 1.87)	Moderate	0.41 (0.09 to 1.87)	Moderate	0.44 (0.13 to 1.53)	Moderate

^1^Rated down one level for incoherence.

CalSevMag: Calcium and sevelamer and magnesium; CI: Confidence interval; RR: Risk ratio.

S3 Fig depicts the comparison-adjusted funnel plot using random effect models. The comparison-adjusted funnel plot does not indicate the presence of small study effects.

Additionally, using visual interpretation, we compared RRs and 95% CIs from the consistency and inconsistency models (Table 3). The proximity of the RRs and overlap between 95% CIs were not satisfactory for the comparisons of calcium with sevelamer and iron with sevelamer. We therefore rated down quality of network evidence for incoherence.

### Two-node analysis: Calcium-based phosphate binders versus non-calcium based phosphate binders

S4, S5 and S6 Figs present the results of our conventional meta-analysis of all-cause mortality, cardiovascular mortality and hospitalization. Fifteen studies that randomized patients to calcium versus NCBPBs showed an increase in all-cause mortality with calcium (RR 1.760 [95%CI, 1.21 to 2.56], moderate quality evidence) (S4 Fig). The outcome of cardiovascular mortality was based on five studies and did not prove significant (RR, 2.54 [95% CI, 0.67 to 9.62; low quality of evidence) (S5 Fig). The results of 3 studies suggest higher, although non-significant, hospitalization with calcium than NCBPBs (RR, 1.28 [95% CI,0.94 to 1.74]; moderate quality of evidence) (S6 Fig). S2 Table presents the GRADE evidence profile associated with these results.

## Discussion

### Summary of main results

The results of this NMA provide moderate quality evidence that calcium causes higher rates of mortality versus sevelamer among CKD-MBD patients (NMA RR, 1.89 [95% CI, 1.02to 3.50]). This is consistent with our finding of an increase in mortality with calcium versus NCBPB in general from a conventional meta-analysis, and translates into an absolute increase in mortality of 43 cases per 1000 (95% CI 23 to 80 more). Although not statistically significant, conventional meta-analysis results also suggest an increase in cardiovascular mortality and hospitalization with calcium versus NCBPBs.

### Underlying hypothesis related to the link between type of phosphate binders and the cardiovascular risk

Vascular smooth muscle cells can assume an osteoblast phenotype through phosphorous mediated and non-phosphorous mediated systems [2,62–64]. This leads to an increase in vascular stiffness, afterload, and promotes left ventricular hypertrophy [2, 60–62]. Elevated calcium, parathyroid hormone and parathyroid hormone-like peptides provoke and promote the abnormal calcification process and cardiovascular diseases[63, 64]. Calcium-based phosphate binders can cause hypercalcemia and contribute to cardiovascular calcification[13]. This condition eventually leads to cardiovascular mortality which is the leading cause of death in patients with CKD[65, 66]. Recently, a systematic review of the 11 RCTs including 1501 patients found that lanthanum reduced the incidence of hypercalcemia relative to calcium [67].

Comparative effectiveness studies of NCBPBs have used calcium as the comparator [16]. While our meta-analysis, and that of Jamal et el. suggests increased all-cause mortality with calcium compared with NCBPBs [16], this apparent benefit may be due to harmful effects of calcium, rather than beneficial effects with NCBPBs. The harmful effect of calcium is consistent with the role of calcium in the pathophysiology of vascular calcification [63, 64] and is also supported by studies in the general population suggesting increased cardiovascular risk with higher levels of calcium exposure [68].

Whether the increase in mortality with calcium versus NCBPBs represents a harmful effect of calcium versus no treatment for hyperphosphatemia, or a beneficial effect of NCBPBs, should ideally be informed by trials of NCBPBs versus placebo, no treatment, or a phosphorus restricted diet. Unfortunately, our NMA provides little information in this regard: although we were able to adduce estimates, the confidence intervals are sufficiently wide as to be uninformative (Table 3).

Thus, additional evidence is required to address this issue. Potential benefits of NCBPBs may be particularly difficult to prove in the context of a moderate-sized randomized trial. Since vascular medial calcification is a result of cellular differentiation, the degree to which it is reversible is likely limited. Long nocturnal hemodialysis, for example, provides excellent biochemical control and can induce negative calcium and phosphorus balance, but does not consistently promote regression of vascular calcification [69–71]. Therefore, in clinical trials with relatively short follow-up, and high attrition rates, one might not expect to see significant reversal of established vascular calcification or major effects on cardiovascular and all-cause mortality.

### Consistency of our findings with the existing evidence

Our finding that calcium leads to increased mortality versus NCBPBs is congruent with results reported with previous systematic reviews using head-to-head comparisons [16]. Although there are strong associations between calcium, phosphate and parathyroid hormone with survival and cardiovascular events, these measures may simply represent vigilance of care and are not necessarily causally related to these outcomes [72, 73]. A recent systematic review examined the correlation between CKD-MBD biochemical markers and mortality and indicated a significant negative correlation between parathyroid hormone and all-cause mortality [74]. Nevertheless, the correlation between serum calcium and phosphorus concentration and mortality did not prove significant [74].

### Strengths and limitations of this study

Strengths of our review include explicit eligibility criteria, a comprehensive search, and independent duplicate assessment of eligibility. Our analysis incorporates the latest developments in NMA statistical analysis and we applied the recently developed GRADE approach to NMA that included assessment of transitivity assumptions for indirect evidence as well as coherence for combining direct and indirect evidence. This is the first systematic review and network meta-analysis that includes iron-based phosphate binders.

The main weakness of our study was limited statistical power for a number of comparisons. With the exception of sevelamer, we were unable to establish the impact of individual NCBPBs on all-cause mortality in relation to calcium, nor were we able to inform the impact of any NCBPB on mortality relative to placebo, or phosphorus diet restriction.

As previously mentioned, inadequate follow-up time in some of the trials was another weakness of our data. Overall, the lack of long-term outcome data of patients with CKD-MBD necessitates conduct of large RCTs with longer follow-up. Another option would be observational studies with longer follow-up that capture mortality if long-term RCTs are unfeasible.

## Conclusions and Future Directions

CKD-MBD is a systematic condition defined by an increase in cardiovascular calcifications and bone fragility [75]. A consensus exists regarding the need for CKD-MBD treatment to maintain guideline recommended targets for calcium, phosphorus and parathyroid hormone in the presumption that meeting these targets will improve quality and quantity of life [76].

Our systematic review suggests that calcium, as compared to NCBPBs in general and sevalamer in particular, increases all-cause mortality among CKD-MBD patients. Future studies should start at earlier stages of CKD, before irreversible calcification is established.

The finding of higher mortality with calcium than alternative phosphate binders, and the possibility that this increase in mortality represents an adverse effect of calcium rather than any benefit with NCBPB, raises serious questions about the advisability, and perhaps the ethical acceptability, of calcium administration in patients with CKD-MBD.

## Supporting Information

S1 Fig Risk of bias assessment; outcome: all-cause mortality. Low risk of bias for missing data and selective reporting in about 75% of the trials.
(TIF)

S2 Fig Contribution plot of phosphate binders for CKD-MBD; outcome: all-cause mortality. (TIF)

S3 Fig The comparison-adjusted funnel plot for the phosphate binder network; outcome: all-cause mortality. (TIF)

S4 Fig Forest plot, calcium-based versus non-calcium-based phosphate binders; outcome: all-cause mortality. (TIF)

S5 Fig Forest plot, calcium based vs. non-calcium based phosphate binders; outcome: cardiovascular mortality. (TIF)

S6 Fig Forest plot, calcium based vs. non-calcium based phosphate binders; outcome: hospitalization. (TIF)

S1 File PRISMA NMA checklist. (DOCX)

S2 File Search strategies. (DOCX)

S1 Table Study Characteristics. (DOCX)

S2 Table GRADE quality assessments of direct evidence per pairwise treatment comparison for all-cause mortality, cardiovascular mortality and hospitalization due to any reason. (DOCX)

## Abbreviations

CBPB: Calcium based phosphate binders
CI: Confidence intervals
CKD-MBD: Chronic kidney disease-mineral and bone disorders
GRADE: Grading of Recommendations, Assessment, Development and Evaluation
NCBPB: Non-calcium based phosphate binders
NMA: Network meta-analysis
RCT: Randomized controlled trail
OR: odds ratio
