# Supplementary figures and images for: Comparative Effectiveness of Phosphate Binders in Patients with Chronic Kidney Disease: A Systematic Review and Network Meta-Analysis
Source: PLoS One. 2016 Jun 8;11(6):e0156891. doi: 10.1371/journal.pone.0156891 (PMC4898688; doi:10.1371/journal.pone.0156891)

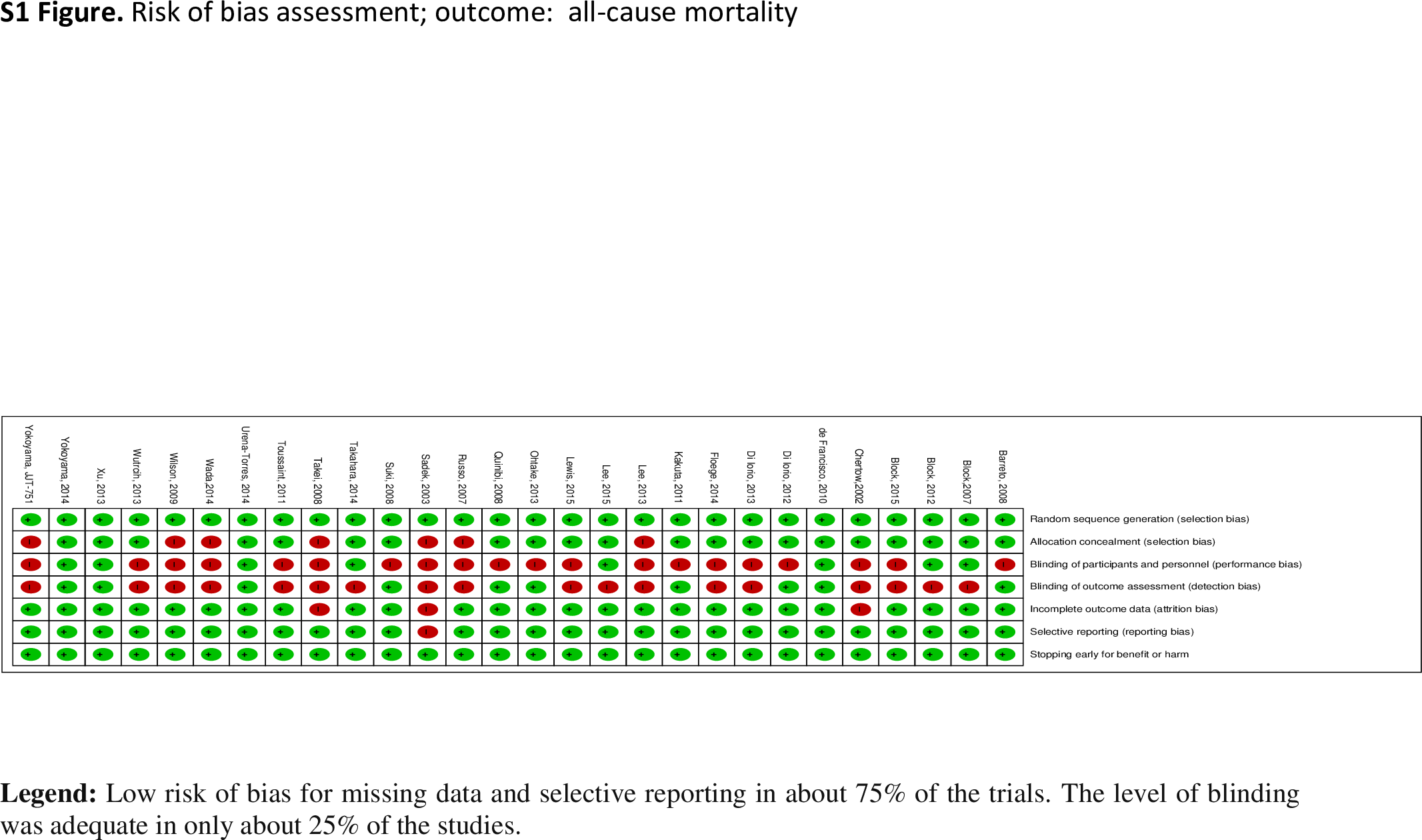

Supplement: S1 Fig — Low risk of bias for missing data and selective reporting in about 75% of the trials. (TIF) [file pone.0156891.s001.tif]

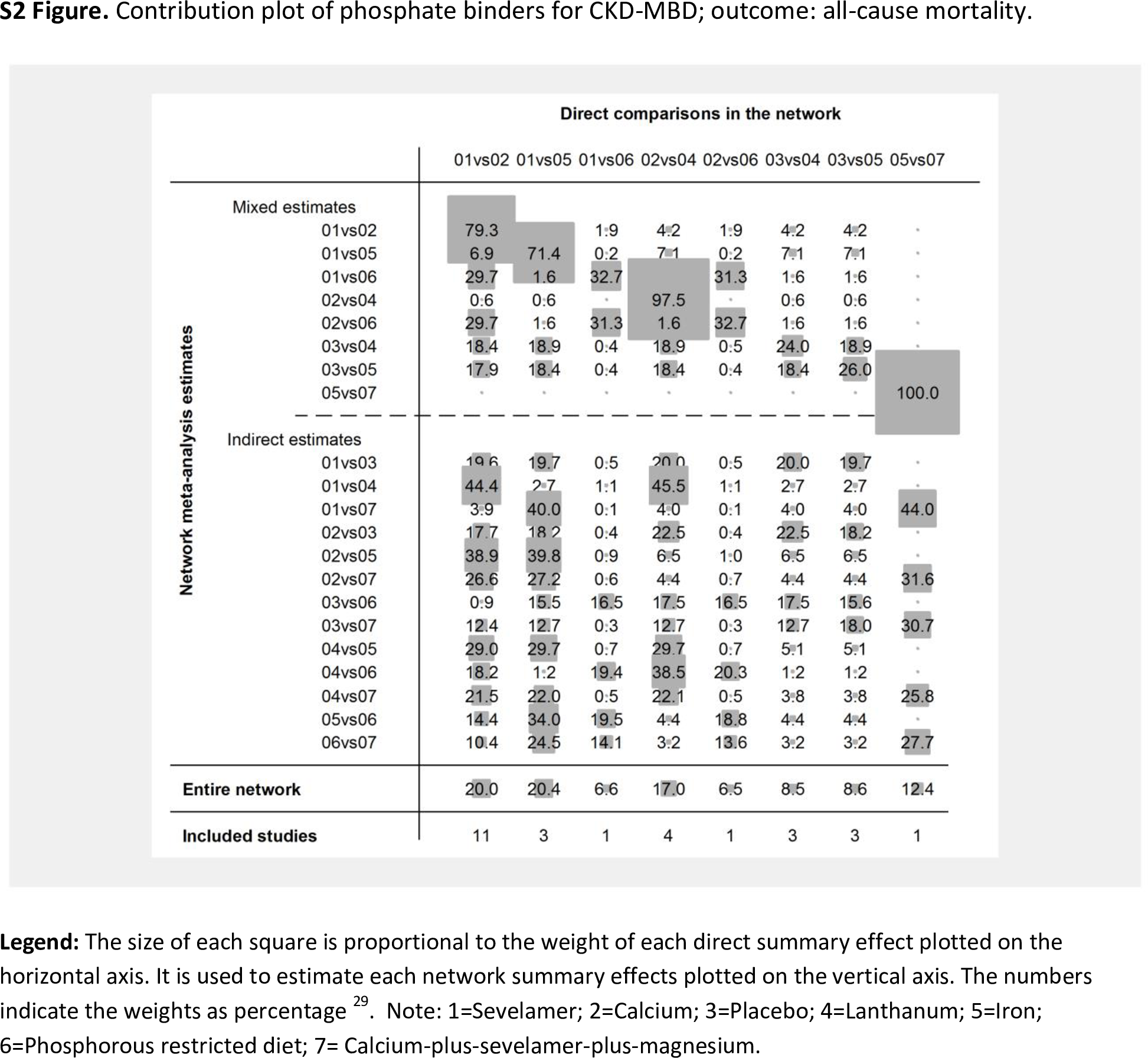

Supplement: S2 Fig — (TIF) [file pone.0156891.s002.tif]

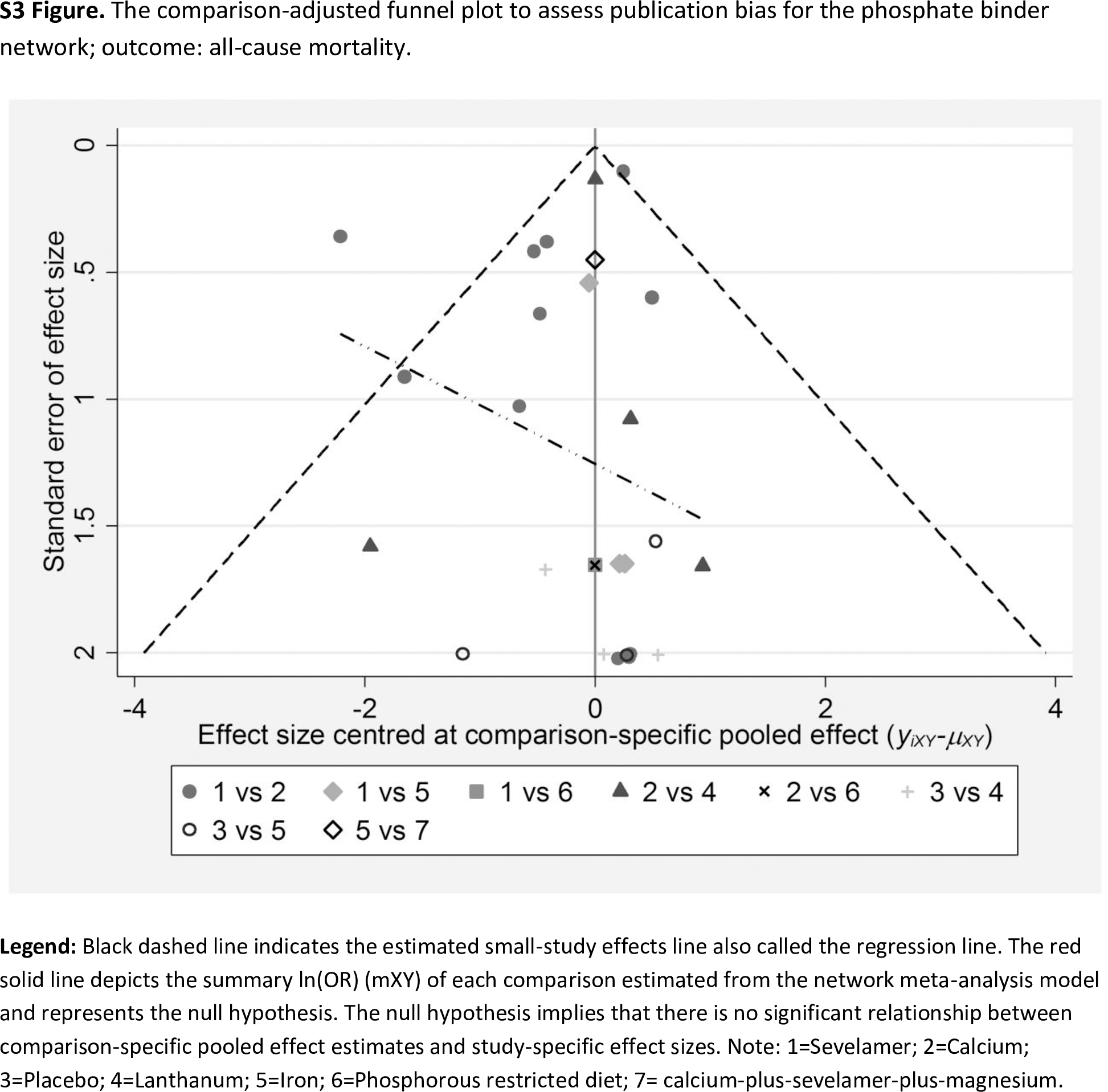

Supplement: S3 Fig — (TIF) [file pone.0156891.s003.tif]

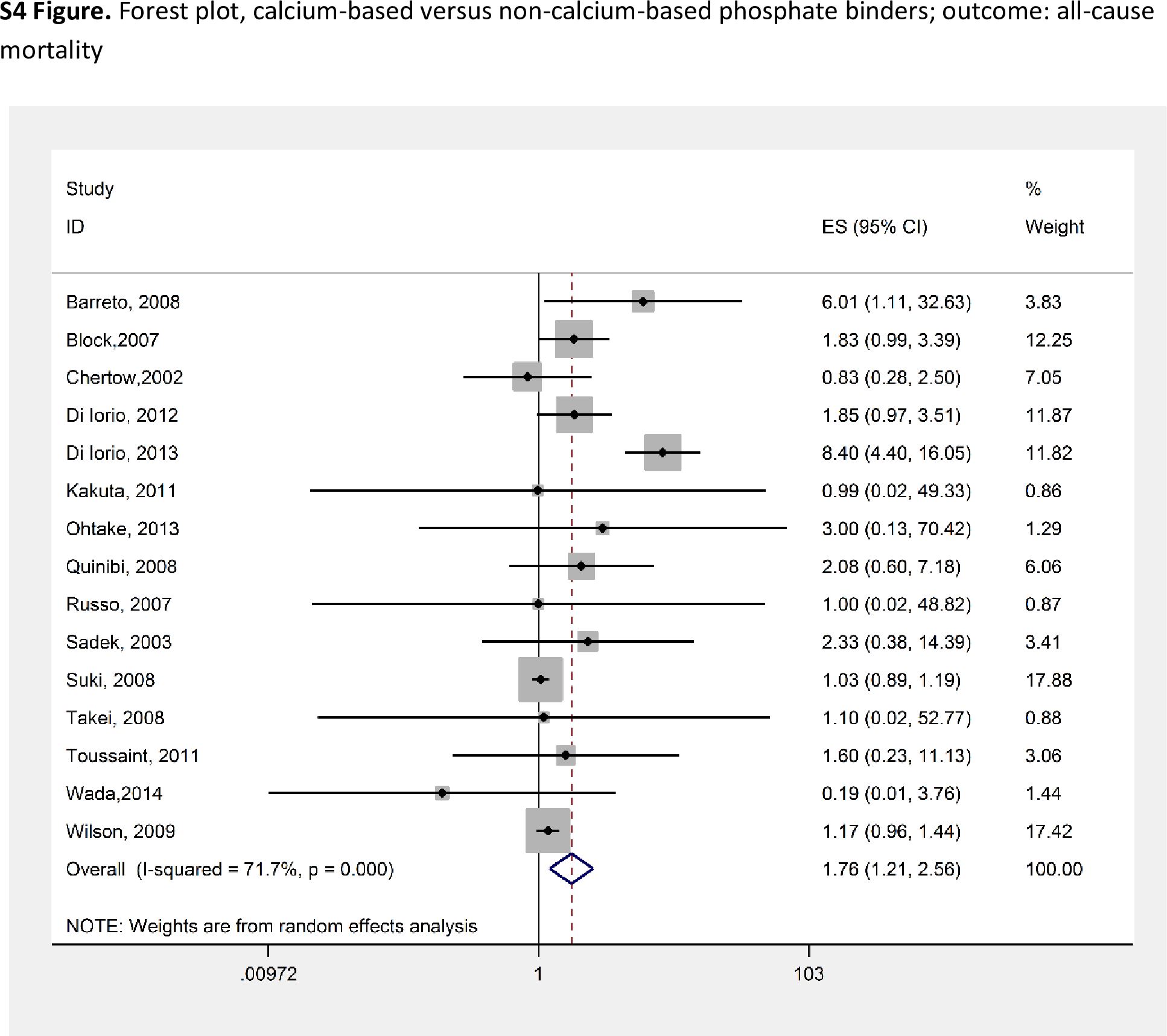

Supplement: S4 Fig — (TIF) [file pone.0156891.s004.tif]

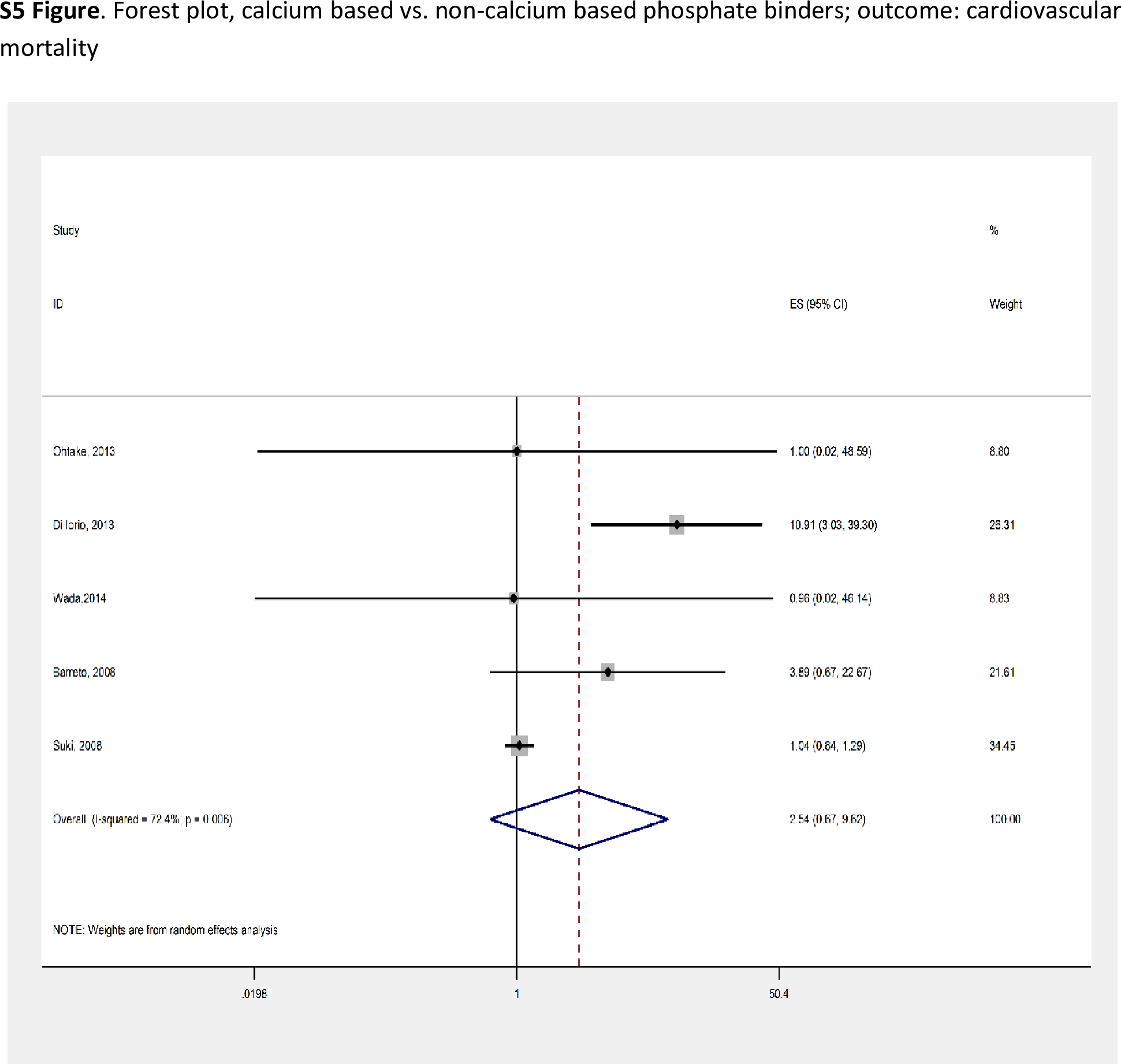

Supplement: S5 Fig — (TIF) [file pone.0156891.s005.tif]

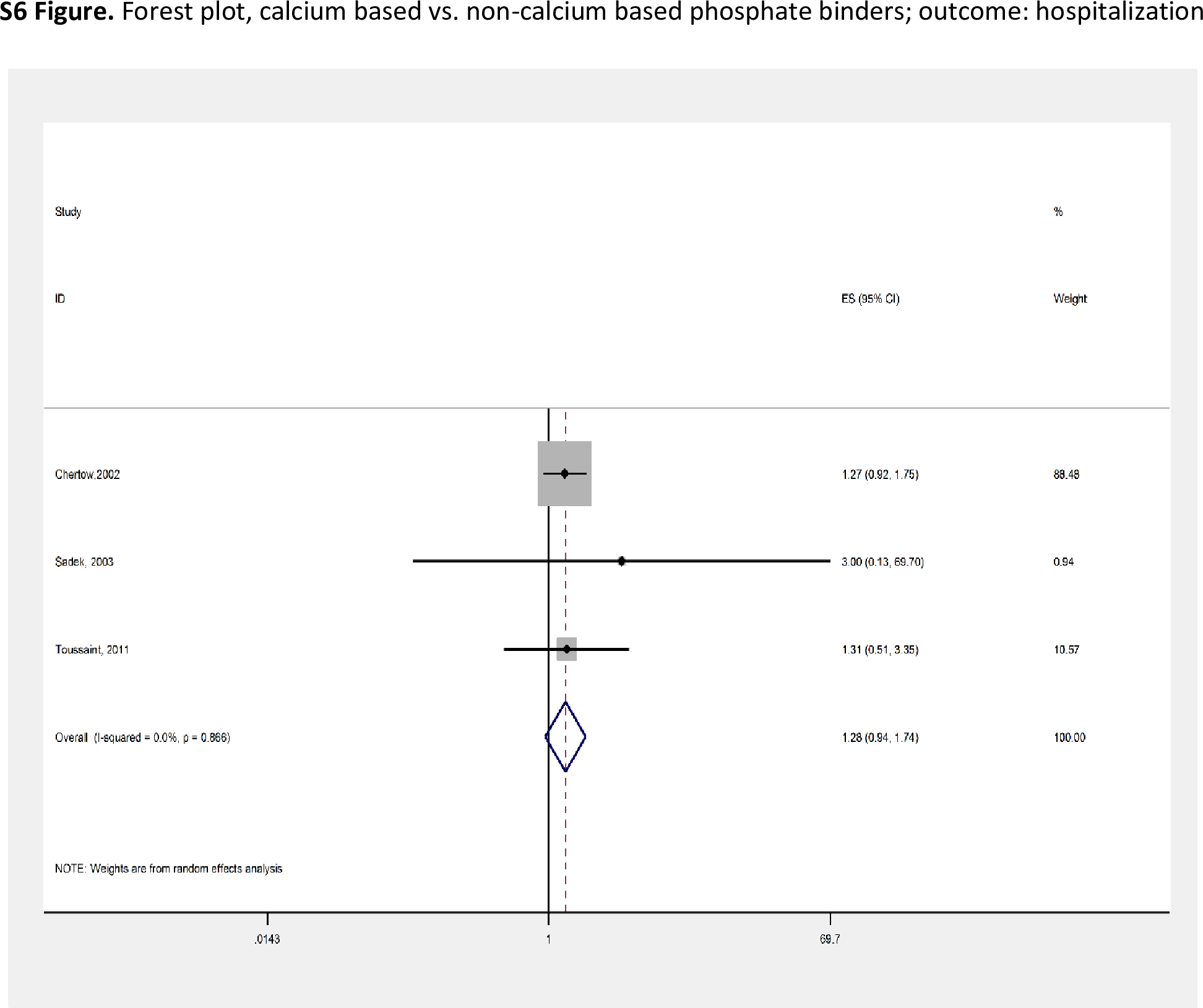

Supplement: S6 Fig — (TIF) [file pone.0156891.s006.tif]
