# Supplementary material for: Comparative Effectiveness of Phosphate Binders in Patients with Chronic Kidney Disease: A Systematic Review and Network Meta-Analysis
Source: PLoS One. 2016 Jun 8;11(6):e0156891. doi: 10.1371/journal.pone.0156891 (PMC4898688; doi:10.1371/journal.pone.0156891)
Supplement: S2 File — (DOCX) [file pone.0156891.s008.docx]

**S1 File eSearch strategies:**

MEDLINE OVID

| 1. | (((kidney* or nephro* or renal or home or peritoneal or intermittent or chronic or extracorporeal or ambulatory) adj2 (haemodialys* or hemodialys* or dialys*)) or hemorenodialysis or hemodialyse or CAPD).ti,ab. |
| --- | --- |
| 2. | renal dialysis/ or hemodialysis, home/ or peritoneal dialysis/ or peritoneal dialysis, continuous ambulatory/ |
| 3. | renal insufficiency, chronic/ or kidney failure, chronic/ |
| 4. | (((chronic or "end-stage" or "end stage") adj3 (kidney* or renal or nephro*) adj3 (insufficien* or disease*)) or esrd).ti,ab. |
| 5. | renal osteodystrophy/ or ((renal or kidney* or nephro*) adj2 (osteodystroph* or ricket*)).mp. |
| 6. | azotemia/ or azotemi*.mp |
| 7. | uremia/ or uremi*.mp. |
| 8. | 1 or 2 or 3 or 4 or 5 or 6 or 7 |
| 9. | controlled clinical trial.pt. or controlled clinical trials as topic/ or meta analysis.pt. or meta analysis as topic/ or multicentre study.pt. or multicenter studies as topic/ or randomized controlled trial.pt. or randomized controlled trials as topic/ or pragmatic clinical trial.pt. or Pragmatic Clinical Trials as Topic/ or ((preference or practical or pragmatic or "real world" or naturalistic) adj5 trial*).ti,ab. or Comparative Effectiveness Research/ or ((comparative adj2 effectiveness) or (CER adj5 (research* or method* or framework* or compari* or statement*))).ti,ab. or ((singl: or doubl: or tripl: or trebl:) and (mask: or blind:)).ti,ab. or ((random: adj5 trial:) or rct or rcts).ti,ab. |
| 10. | calcium/ or (calc* or calc* acet* or Calc* acet* or Cal* car* or Cal*Car).mp. [mp=title, abstract, original title, name of substance word, subject heading word, keyword heading word, protocol supplementary concept word, rare disease supplementary concept word, unique identifier] |
| 11. | (phosphate binders or phosphate lowering agent).mp. [mp=title, abstract, original title, name of substance word, subject heading word, keyword heading word, protocol supplementary concept word, rare disease supplementary concept word, unique identifier] |
| 12. | (lanthanum or lanthanum carbonate).mp. [mp=title, abstract, original title, name of substance word, subject heading word, keyword heading word, protocol supplementary concept word, rare disease supplementary concept word, unique identifier] |
| 13. | (sevelamer or sevela*).mp. [mp=title, abstract, original title, name of substance word, subject heading word, keyword heading word, protocol supplementary concept word, rare disease supplementary concept word, unique identifier] |
| 14. | (iron or ferrous citrate).mp. [mp=title, abstract, original title, name of substance word, subject heading word, keyword heading word, protocol supplementary concept word, rare disease supplementary concept word, unique identifier] |
| 14. | 10 or 11 or 12 or 13 or 14 |
| 15. | 8 and 9 and 14 |
| 16. | limit 25 to yr="2013 -Current" |

EMBASE OVID

| 1. | (((kidney* or nephro* or renal or home or peritoneal or intermittent or chronic or extracorporeal or ambulatory) adj2 (haemodialys* or hemodialys* or dialys*)) or hemorenodialysis or hemodialyse or CAPD).ti,ab. |
| --- | --- |
| 2. | renal dialysis/ or hemodialysis, home/ or peritoneal dialysis/ or peritoneal dialysis, continuous ambulatory/ |
| 3. | renal insufficiency, chronic/ or kidney failure, chronic/ |
| 4. | (((chronic or "end-stage" or "end stage") adj3 (kidney* or renal or nephro*) adj3 (insufficien* or disease*)) or esrd).ti,ab. |
| 5. | renal osteodystrophy/ or ((renal or kidney* or nephro*) adj2 (osteodystroph* or ricket*)).mp. |
| 6. | azotemia/ or azotemi*.mp |
| 7. | uremia/ or uremi*.mp. |
| 8. | 1 or 2 or 3 or 4 or 5 or 6 or 7 |
| 9. | controlled clinical trial.pt. or controlled clinical trials as topic/ or meta analysis.pt. or meta analysis as topic/ or multicentre study.pt. or multicenter studies as topic/ or randomized controlled trial.pt. or randomized controlled trials as topic/ or pragmatic clinical trial.pt. or Pragmatic Clinical Trials as Topic/ or ((preference or practical or pragmatic or "real world" or naturalistic) adj5 trial*).ti,ab. or Comparative Effectiveness Research/ or ((comparative adj2 effectiveness) or (CER adj5 (research* or method* or framework* or compari* or statement*))).ti,ab. or ((singl: or doubl: or tripl: or trebl:) and (mask: or blind:)).ti,ab. or ((random: adj5 trial:) or rct or rcts).ti,ab. |
| 10. | calcium/ or (calc* or calc* acet* or Calc* acet* or Cal* car* or Cal*Car).mp. [mp=title, abstract, original title, name of substance word, subject heading word, keyword heading word, protocol supplementary concept word, rare disease supplementary concept word, unique identifier] |
| 11. | (phosphate binders or phosphate lowering agent).mp. [mp=title, abstract, original title, name of substance word, subject heading word, keyword heading word, protocol supplementary concept word, rare disease supplementary concept word, unique identifier] |
| 12. | (lanthanum or lanthanum carbonate).mp. [mp=title, abstract, original title, name of substance word, subject heading word, keyword heading word, protocol supplementary concept word, rare disease supplementary concept word, unique identifier] |
| 13. | (sevelamer or sevela*).mp. [mp=title, abstract, original title, name of substance word, subject heading word, keyword heading word, protocol supplementary concept word, rare disease supplementary concept word, unique identifier] |
| 14. | (iron or ferrous citrate).mp. [mp=title, abstract, original title, name of substance word, subject heading word, keyword heading word, protocol supplementary concept word, rare disease supplementary concept word, unique identifier] |
| 14. | 10 or 11 or 12 or 13 or 14 |
| 15. | 8 and 9 and 14 |
| 16. | limit 25 to yr="2013 -Current" |

EBM Reviews - Cochrane Central Register of Controlled Trials

| 1 | chronic kidney disease:ti,ab,kw | 4911 |
| --- | --- | --- |
| 2 | Phosphate binders | 266 |
| 3 | randomized controlled trials | 571289 |
| 4 | 1 and 2 and 3 | 82 |
