# Supplementary material for: Comparative Effectiveness of Phosphate Binders in Patients with Chronic Kidney Disease: A Systematic Review and Network Meta-Analysis
Source: PLoS One. 2016 Jun 8;11(6):e0156891. doi: 10.1371/journal.pone.0156891 (PMC4898688; doi:10.1371/journal.pone.0156891)
Supplement: S1 Table — (DOCX) [file pone.0156891.s009.docx]

**S1 Table.** Study Characteristics

| Study, Year (Reference) | Country | Randomly assigned patients, n | Number of arms | Women, % | Age, y (SD) | Stage of CKD | Comparison | Diabetes, n (%) | Hypertension, n (%) | Follow-up duration in months |
| --- | --- | --- | --- | --- | --- | --- | --- | --- | --- | --- |
| Chertow et al, 2002[^35^](#_ENREF_35) | United States, Austria and Germany | 99  101 | 2 | 70, 35% | 57 (14)  56 (16) | Stage 5D | Sevelamer vs. calcium | 32%  33% | 86%  83% | 12 |
| Sadek et al, 2003[^47^](#_ENREF_47) | France | 21  21 | 2 | - | - | Stage 5D | Sevelamer vs. calcium | - | - | 5 |
| Block et al, 2007 [^33^](#_ENREF_33) | United States and Italy | 60  67 | 2 | 42%  36% | 56 (14)  58 (14) | Stage 5D | Sevelamer vs. calcium | 60%  56% | 95%  98% | 60 |
| Russo et al, 2007 [^46^](#_ENREF_46) | Italy | 30  30  30 | 3 | 3 (10%)  5 (16%) | 55 (13)  54 (12) | Non-dialysis | Sevelamer vs. calcium vs. phosphorus restricted diet | - | - | 24 |
| Barreto et al, 2008 [^31^](#_ENREF_31) | Brazil | 52  49 | 2 | 34%  30% | 47 (13)  47 (14) | Stage 5D | Sevelamer vs. calcium | 15%  13% | 66%  73% | 12 |
| Qunibi et al, 2008 [^45^](#_ENREF_45) | United States | 100  103 | 2 | 54%  42% | 60 (12)  58 (12) | Stage 5D | Sevelamer vs. calcium | 57%  57% | 31%  31% | 12 |
| Suki et al, 2008[^48^](#_ENREF_48) | United States | 1053  1050 | 2 | 479 (45%)  481(48%) | 59 (14)  60 (15) | Stage 5D | Sevelamer vs. calcium | 50%  50% | 33%  33% | 45 |
| Takei et al, 2008[^50^](#_ENREF_50) | Japan | 22  20 | 2 | 50%  45% | 54 (10)  54 (9) | Stage 5D | Sevelamer vs. calcium | 36%  36% | - | 6 |
| Wilson et al, 2009 [^54^](#_ENREF_54) | United States and United Kingdom | 680  674 | 2 | 42%  38% | 54 (14)  60 (14) | Stage 5D | Lanthanum vs.  Standard treatment | 34%  34% | 31%  28% | 24 |
| De Francisco et al,[^34^](#_ENREF_34) 2010[^36^](#_ENREF_36) | Spain, Portugal, Germany, Italy, Romania and Poland | 127  125 | 2 | 49%  47% | 56 (12)  59 (14) | Stage 5D | Sevelamer vs. calcium | 20%  24% | - | 6 |
| Kakuta et al, 2011 [^40^](#_ENREF_40) | Japan | 91  92 | 2 | 43%  49% | 59 (12)  57 (12) | Stage 5D | Sevelamer vs. calcium | - | 60%  64% | 12 |
| Toussaint et al, 2011 [^51^](#_ENREF_51) | Australia | 22  23 | 2 | 45%  26% | 56 (15)  59 (15) | Stage 5D | Lanthanum vs calcium | 32%  35% | 9%  9% | 18 |
| Block et al, 2012[^34^](#_ENREF_34) | United States, Germany and United Kingdom | 57  28  30  30 | 4 | 21%  18%  20%  20% | 65 (12)  70 (10)  66 (12)  68 (12) | Non-dialysis | Placebo vs.  Lanthanum vs.  Sevelamer vs.  Calcium | 58%  57%  53%  57% | 100%  100%  97%  97% | 10 (median follow-up time 249 days) |
| Di Iorio et al, 2012[^37^](#_ENREF_37) | United States and Italy | 232  234 | 2 | 50%  52% | 67 (14)  65 (15) | Stage 5D | Sevelamer vs. calcium | 30%  29% | 78%  81% | 24 |
| Di Iorio et al, 2013 [^38^](#_ENREF_38) | Italy | 121  118 | 2 | 39%  39% | 57 (12)  59 (12) | Non-dialysis | Sevelamer vs. calcium | 27%  29% | 73%  76% | 36 |
| Lee et al, 2013 [^42^](#_ENREF_42) | Korea | 50 | 2 | 45%  63% | 48 (11)  52 (11) | Stage 5D | Lanthanum vs calcium | 30%  17% | 35%  37% | 6 |
| Ohtake et al, 2013 [^44^](#_ENREF_44) | Japan | 26  26 | 2 | 40% | 68 (6) | Stage 5D | Lanthanum vs calcium | 43% | - | 12 |
| Wuthrich et al, 2013 [^55^](#_ENREF_55) | Canada, United States, Romania, and Switzerland | 24  126 | 2 | 58%  37% | 60 (13)  62 (11) | Stage 5D | Sevelamer vs. sucroferric oxyhydroxide | 38%  32% | 67%  75% | 1.5 |
| Xu et al, 2013 [^56^](#_ENREF_56) | China | 115  115 | 2 | 47%  36% | 48 (13)  48 (12) | Stage 5D | Lanthanum vs. Placebo | - | - | 2 |
| Floege et al, 2014[^39^](#_ENREF_39) | United States, Romania, Germany and Switzerland | 349  710 | 2 | 37%  45% | 56 (15)  56 (13) | Stage 5D | Sevelamer vs. sucroferric oxyhydroxide | - | - | 6 |
| Takahara et al, 2014 [^49^](#_ENREF_49) | Japan | 86  55 | 2 | 55%  27% | 61 (11)  62 (13) | Non-dialysis | Lanthanum vs. Placebo | - | - | 2 |
| Urena-Torres et al, 2014 [^52^](#_ENREF_52) | France | 17  12 | 2 | 41%  58% | 66 (15)  69 (13) | Non-dialysis | Lanthanum vs. Placebo | - | - | 3 |
| Wada et al, 2014 [^53^](#_ENREF_53) | Japan | 21  22 | 2 | 23%  21% | 66 (10)  66 (8) | Stage 5D | Lanthanum vs calcium | 100%  100% | - | 12 |
| Yokoyama et al, 2014 [^57^](#_ENREF_57) | Japan and Unites States | 110  115 | 2 | 35%  37% | 62 (10)  60 (11) | Stage 5D | Sevelamer vs JTT-751 | - | - | 3 |
| Yokoyama et al, 2014 [^58^](#_ENREF_58) | Japan and Unites States | 60  30 | 2 | 42%  41% | 65 (10)  65 (14) | Non-dialysis and dialysis | Ferric citrate vs. Placebo | - | - | 3 |
| Block et al, 2015[^32^](#_ENREF_32) | United States, Germany and Spain | 75  74 | 2 | 69%  62% | 66 (12)  64 (14) | Non-dialysis and dialysis | Ferric citrate vs. Placebo | 67%  71% | - | 3 |
| Lee et al, 2015 [^41^](#_ENREF_41) | Taiwan | 36  75  72 | 3 | 37%  43%  31% | 53 (12)  53 (11)  53 (12) | Stage 5D | Ferric citrate vs. Placebo | - | - | 2 |
| Lewis et al, 2015 [^43^](#_ENREF_43) | United States | 292  149 | 2 | 37%  42% | 56 (45-63)  54 (45-63) | Stage 5D | Ferric citrate vs.  Active control (calcium acetate and sevelamer) | - | - | 12 |
