# Supplementary material for: Comparative Effectiveness of Phosphate Binders in Patients with Chronic Kidney Disease: A Systematic Review and Network Meta-Analysis
Source: PLoS One. 2016 Jun 8;11(6):e0156891. doi: 10.1371/journal.pone.0156891 (PMC4898688; doi:10.1371/journal.pone.0156891)
Supplement: S2 Table — (DOCX) [file pone.0156891.s010.docx]

**S2 Table.** GRADE quality assessments of direct evidence per pairwise treatment comparison for all-cause mortality, cardiovascular mortality and hospitalization due to any reason.

| Outcome | Number of studies number of participants | Study Limitations | Precision | Consistency | Directness | Publication bias | Overall quality of evidence | Relative effect estimate^1^; OR (95% CI) |
| --- | --- | --- | --- | --- | --- | --- | --- | --- |
| All-cause mortality | 15;5260 | Not serious | Not serious | Serious  (I^2^, 74.3%) | Not serious | Not serious | Moderate | 1.76 (1.21 to 2.56) |
| Cardiovascular mortality | 5,2765 | Not serious | Serious | Serious  (I^2^, 73.4%) | Not serious | Not serious | Low | 2.54 (0.67 to 9.62) |
| Hospitalization due to any reason | 3; 287 | Not serious | Serious | Not serious  (I^2^, 0%) | Not serious | Not serious | Moderate | 1.28 (0.94 to 1.74) |

**Legend:** Ratings for domains “Study limitations”, “Precision”, “Consistency”, and “Directness” were: Not serious, Serious, or Very serious issues. For the domain “Publication bias”: Not likely or Likely to exist. Reasons are provided when rating down. All direct comparisons begin with a “High” quality rating.^1^We employed random effect models.
